# A Major Latex Protein-Encoding Gene from *Populus simonii × P. nigra* (*PsnMLP328*) Contributes to Defense Responses to Salt and Cadmium Stress

**DOI:** 10.3390/ijms26073350

**Published:** 2025-04-03

**Authors:** Xin Sun, Lei Wang, Shuang Liu, Yao Li, Yao Sun, Qiong Wu, Di Fu

**Affiliations:** Department of Biotechnology, Institute of Advanced Technology, Heilongjiang Academy of Sciences, Harbin 150001, China; sunxin@cau.edu.cn (X.S.);

**Keywords:** major latex protein, *Populus simonii × P. nigra*, salt stress, cadmium stress

## Abstract

Heavy metal pollution and soil salinization harm human health and the environment. Phytoremediation is a widely accepted soil decontamination method, with woody plants being particularly effective due to their large biomass and extensive root systems. In this study, we identified and cloned *PsnMLP328* from *Populus simonii × P. nigra* and demonstrated its role in mitigating salt and cadmium stress. *PsnMLP328* expression was up-regulated under both stress conditions, and its overexpression in tobacco enhanced resistance to these stresses, albeit through distinct mechanisms. Transgenic plants exhibited increased Cd^2^^+^ uptake and a higher biomass, alleviating Cd^2^^+^-induced growth inhibition. Additionally, *PsnMLP328* boosted proline content, chlorophyll levels, and antioxidative enzyme activities (POD, SOD) under Cd^2^^+^ stress, likely by protecting cells from oxidative damage. Expression analysis revealed that *PsnMLP328* down-regulated the cadmium transporter *Nramp2* while up-regulating *YSL2* (another cadmium transporter) and potassium channels (*AKT1* and *AKT2/3*), suggesting its role in modulating K^+^ and Cd^2+^ homeostasis. These findings indicate that *PsnMLP328* enhances tobacco resistance to salt and cadmium stress, particularly the latter. This study is the first to elucidate the function of poplar *MLP* family genes under salt and cadmium stress, advancing our understanding of *MLP* gene roles in heavy metal stress and offering new insights for remediating salinized and heavy metal-contaminated soils.

## 1. Introduction

In recent years, industrialization has led to a decrease in cultivated land and worsening soil issues, posing significant challenges for a growing population [1]. Key soil problems include soil salinization and heavy metal pollution, with salinization affecting over 40% of cultivated land [2]. The primary sources of salt in soil include weathering of the soil surface and salts from the ocean carried by wind and rain, as well as manufactured factors such as the excessive irrigation of farmed land. Excessive levels of salt in the soil can cause wilting from dehydration, delayed plant growth, poor seed germination, and, in extreme situations, death [2,3]. Salinization of cultivable land can drastically reduce crop yields, severely hindering agricultural development. This impact varies across plants, as they exhibit different levels of growth inhibition under salt stress, reflecting their varying tolerances [4].

The effects of salt stress on plants primarily stem from osmotic stress and ion toxicity [5]. Plants have developed a series of physiological, biochemical, and molecular regulatory mechanisms to cope with and to resist salt stress. These mechanisms include the perception of salt stress; the selective absorption, accumulation, or exclusion of ions; the compartmentalization of harmful ions through membrane systems, and the induction of stress-tolerant gene expression, among others [6]. In addition to signal regulation, plant hormones such as abscisic acid (ABA), jasmonic acid (JA), ethylene (ET), and auxins not only play roles in plant growth and development but also participate in responses to biotic and abiotic stresses. Furthermore, plants accumulate protective metabolites (e.g., polyols, betaine, trehalose, proline, soluble sugars), free unsaturated fatty acids, and phosphatidylinositol to mitigate the oxidative damage and osmotic pressure caused by salt stress [7].

In addition to salinization, heavy metals including lead, cadmium, and mercury have received considerable research attention [8,9]. Cadmium, the most hazardous and toxic heavy metal, can have a harmful effect on plants even at low concentrations [10]. Soil cadmium is derived from human and natural sources. Geological weathering of rocks is the main natural source of Cd pollutants, whereas major anthropogenic sources include agrochemicals, manufacturing, automobile emissions, irrigation wastewater, smelting and mining, and so on [11]. Cadmium can accumulate in plants and has a half-life of 10–30 years. Excessive cadmium uptake by plant roots can cause browning of the root system, decreased root length and dry weight, changes in root morphology, decreased root uptake capacity, cellular damage, and, eventually, root mortality [11,12,13]. Cadmium in crops can endanger human health, and consuming food with high levels of heavy metals over time can damage the human nervous system, internal organs, and bones [14,15].

Plants employ multiple mechanisms to manage Cd stress. Cd stress induces reactive oxygen species (ROS) accumulation, leading to oxidative damage. To counteract this, plants activate their antioxidative systems by enhancing the accumulation of antioxidative enzymes (e.g., SOD, POD, CAT) and non-enzymatic antioxidants (e.g., GSH, ascorbic acid), thereby protecting cells from oxidative damage [16]. Additionally, plants synthesize phytochelatins (PCs) and metallothioneins (MTs) to chelate Cd ions, reducing Cd toxicity. Heavy metal-transport proteins, such as ZIP, NRAMP, HMA, and ABC, play crucial roles in Cd uptake, transport, and efflux, minimizing Cd-induced damage [17]. Remediation of polluted soil is one of the most essential approaches for conserving cultivated land since the area of cultivated land is limited. Since plants can be regulated through various mechanisms to adapt to Cd stress, phytoremediation is considered an economical and sustainable method for remediating Cd-contaminated soils [18,19,20,21,22].

*Populus simonii × P.* nigra is a fast-growing species widely used for greening and afforestation. It exhibits strong environmental adaptability, with notable resistance to cold, drought, and poor soil conditions, as well as moderate salt tolerance. Additionally, it serves as a valuable model for studying poplar physiology. Compared with herbaceous plants, *Populus simonii × P. nigra* possesses a greater biomass and a more extensive root system [23,24]. Its roots can accumulate higher levels of salt and heavy metal ions, effectively removing or stabilizing toxic ions in contaminated soils, making it a highly promising candidate for bioremediation [25,26].

Major latex proteins have a Bet_v_1 domain, which was first discovered in 1985 in opium poppies and later found in several model plants, including cotton, tomato, apple, *Arabidopsis thaliana*, and others [27,28]. The Bet_v_1-domain-containing family includes MLPs, Bet_v_1, and pathogenesis-related protein 10 (PR-10). Research on Bet_v_1 and PR-10 has been conducted for some time, primarily from a medical perspective, due to their roles as allergens in various plants. In contrast, studies on MLPs are fewer and emerged relatively recently [29]. It has been demonstrated that *MLP* family members are able to react to a range of phytohormones, which are crucial for plant growth and development, and have an impact on how plants react to biotic and abiotic stressors from the environment [30,31]. MLPs can indirectly improve plant disease resistance by activating *PR* genes, acting in plant SAR, and producing flavonoids [32,33]. Furthermore, there has been an increasing emphasis on the roles of *MLP*s in the plant abiotic stress response. For instance, Wang et al. found that *MLP43* regulates drought resistance in *Arabidopsis* through the ABA signaling pathway [34], while Liu et al. found that *NtMLP423* regulates tobacco’s ability to tolerate the cold [35]. In addition, salt stress has been shown to trigger certain *MLP* homolog genes, which can modulate plant salt tolerance. Ethylene response factor 76 (ERF76) is a crucial transcription factor that plays a key role in abiotic stress responses. It enhances plant tolerance to drought, high salinity, and low-temperature stress by regulating the expression of downstream stress-related genes [36]. The poplar transcription factor PsnERF76 can bind to PsnMLP328, as we showed in our earlier study (unpublished data), indicating that *PsnMLP328* may also regulate poplar’s abiotic tolerance. As mentioned before, *Populus simonii × P. nigra* exhibits only moderate salt tolerance and struggles to withstand high levels of environmental stress. With increasing soil salinization, metal pollution, and the rapid loss of arable land, enhancing its stress tolerance is crucial for ecological restoration, afforestation, and biomass energy production.

The aim of this study is to investigate the function and potential molecular mechanisms of *PsnMLP328* in response to salt and cadmium stress in plants, using tobacco as a model. Understanding its function and mechanisms could enable the enhancement of salt and Cd stress tolerance in poplar, ultimately facilitating its use in phytoremediation and afforestation.

## 2. Results

### 2.1. Cloning and Sequence Analysis of PsnMLP328

In our previous study, a 326 bp partial gene fragment was isolated utilizing a yeast two-hybrid system. BlastX comparison revealed that the fragment included the typical conserved sequence of a major latex protein; hence, the gene was called *PsnMLP328*. The full-length cDNA of *PsnMLP328* was amplified by RT-PCR using the cDNA of leaves of *Populus simonii × P. nigra* as template, and a band of about 600 bp was obtained. Using an ORF program, it was predicted that the 5′ UTR of the gene was 113 bp and the 3′ UTR was 34 bp. The open reading frame was 453 bp, coding for 150 amino acids—of which Val was the most abundant, accounting for 12% of the total number of amino acids—with 25 negatively charged residues (Asp + Glu) and 17 positively charged residues (Arg + Lys). The molecular weight of the protein encoded by *PsnMLP328* was 16.77 kDa, the theoretical isoelectric point was 5.09, and the instability coefficient was 21.32, which classified it as a stable protein (Table S1). Conserved region prediction showed that PsnMLP328 belonged to the Bet_v_1 superfamily. Subcellular localization prediction indicated that PsnMLP328 would be localized in the cytoplasm. In addition, PsnMLP328 has two Casein kinase II phosphorylation sites, two N-glycosylation sites and three Protein kinase C phosphorylation sites (Table 1).

The results of signal-peptide prediction and transmembrane structure analysis showed that PsnMLP328 has neither a signal peptide nor a transmembrane structure. The phylogenetic analysis results showed that PsnMLP328 was closer to *Populus tremula* and more distant from *Eucalyptus megacephalus* and the cocoa tree (Figure S1).

### 2.2. Response of PsnMLP328 to Salt Stress and Cadmium Stress

In previous studies, it was shown that *AtMLP423*, the Arabidopsis homolog of *PsnMLP328*, functions to regulate abiotic stress in Arabidopsis. We hypothesized that *PsnMLP328* is probably also involved in the response of poplar to abiotic stress; therefore, we examined the expression of *PsnMLP328* at different time points after salt stress and cadmium stress, respectively.

**Figure 1:**
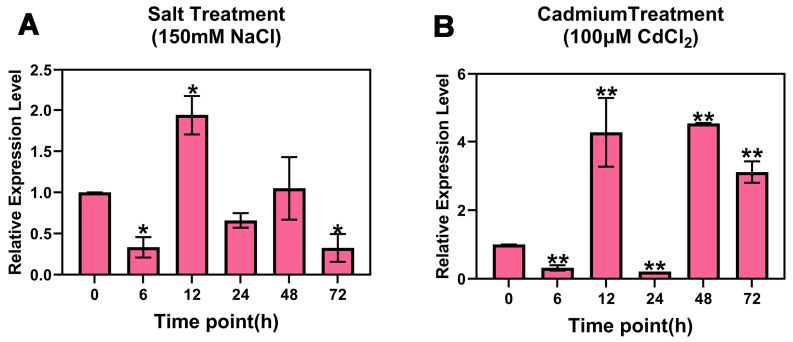
Expression of *PsnMLP328* in poplar. (**A**) Expression of *PsnMLP328* under salt stress. (**B**) Expression of *PsnMLP328* under cadmium stress. * indicates a *p*-value < 0.05; ** indicates a *p*-value < 0.01.

The results showed that the expression of *PsnMLP328* presented a trend of first decreasing and then increasing and decreasing again under salt stress, which was significantly lower than that of the control condition at the 6th hour of stress and significantly higher than that of the control at the 12th hour. Subsequently, with prolongation of the stress time, the expression of *PsnMLP328* gradually decreased (Figure 1A). The trend for *PsnMLP328* expression under cadmium stress was similar but slightly different from that under salt stress. It was also significantly down-regulated at the 6th hour and then significantly up-regulated at the 12th hour, but it decreased to its lowest level at the 24th hour and then continued to increase to the 48th hour, followed by a gradual decrease, and the expression at all time points was significantly different from that of the control (Figure 1B). The results demonstrate that *PsnMLP328* plays a role in poplar's response to both salt and cadmium stress, though potentially through distinct molecular regulatory mechanisms.

### 2.3. Overexpression of PsnMLP328 Can Improve Resistance in Transgenic Tobacco Under Salt and Cadmium Stress

**Figure 2:**
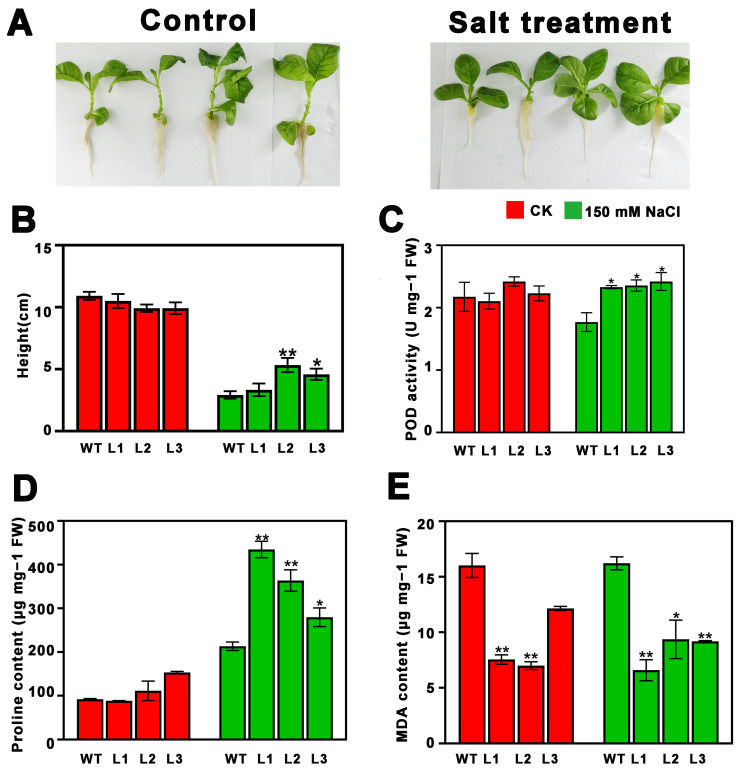
Phenotypic analysis and physiological index detection of transgenic tobacco plants under salt stress. (**A**) Phenotypic analysis of transgenic tobacco plants under salt stress (150 mM NaCl). (**B**) Plant height of WT and transgenic tobacco plants. (**C**) POD activity of WT and transgenic tobacco plants. (**D**) Proline content of WT and transgenic tobacco plants. (**E**) MDA content of WT and transgenic tobacco plants. WT: wild-type; L1–L3: different transgenic lines; * indicates a *p*-value < 0.05; ** indicates a *p*-value < 0.01.

In order to investigate the function of *PsnMLP328* in response to salt and cadmium stress, an overexpression vector of *PsnMLP328* was constructed and transformed into tobacco, and the function of *PsnMLP328* was verified under salt and cadmium stress (Figure S2). Compared with poplar, tobacco is a widely used model plant in research due to its short growth cycle, high genetic transformation efficiency, and ease of laboratory manipulation. Therefore, tobacco was selected as the experimental subject in this study. Plant height did not significantly differ between transgenic and non-transgenic plants in the control setting. However, under salt conditions, the heights of all plants were significantly lower than those under the control condition, and the heights of transgenic tobacco that overexpressed *PsnMLP328* were higher than those of WT (wild type) lines (Figure 2A,B).

Under both control and salt stress conditions, the trans-*PsnMLP328* lines’ MDA content was much lower than that of the WT lines; conversely, the transgenic lines’ proline content was higher than that of the WT lines. The transgenic lines and the WT lines did not significantly differ in their POD activity under control conditions (Figure 2C–E). However, under salt stress conditions, the POD activity of all the transgenic lines was significantly higher than that of the WT lines (Figure 2C–E). All the above results could indicate that the tobacco lines overexpressing *PsnMLP328* were more tolerant to salt stress than the WT lines.

**Figure 3:**
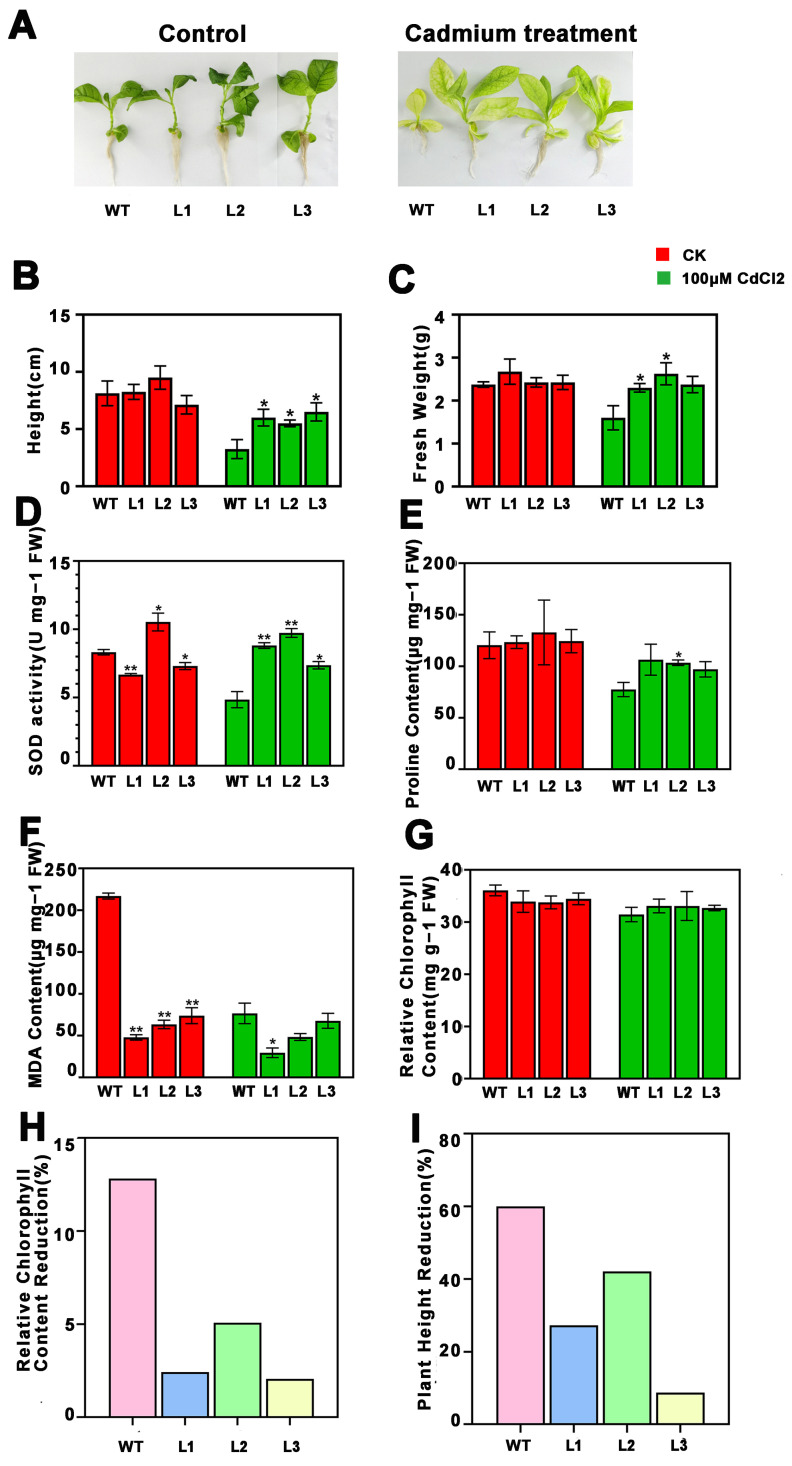
Phenotypic analysis and physiological index detection of transgenic tobacco plants under cadmium stress. (**A**) Phenotypic analysis of transgenic tobacco plants under cadmium stress (100 μM CdCl_2_). (**B**) Plant height of WT and transgenic tobacco plants. (**C**) Fresh weight of WT and transgenic tobacco plants. (**D**) SOD activity in WT and transgenic tobacco plants. (**E**) Proline content of WT and transgenic tobacco plants. (**F**) MDA content of WT and transgenic tobacco plants. (**G**) Relative chlorophyll content of WT and transgenic tobacco plants. (**H**) Relative chlorophyll content reduction in WT and transgenic tobacco plants. (**I**) Plant height reduction in WT and transgenic tobacco plants. WT: wild-type; L1–L3: different transgenic lines; * indicates a *p*-value < 0.05; ** indicates a *p*-value < 0.01.

**Figure 4:**
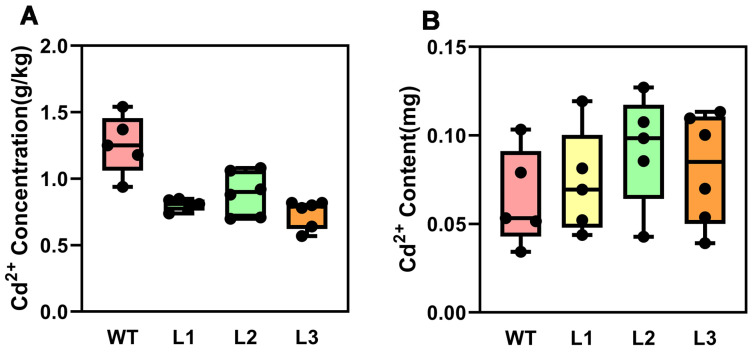
Cd^2+^ concentration and content in different tobacco lines under cadmium stress. (**A**) Cd^2+^ concentration in WT and transgenic tobacco plants under cadmium stress. (**B**) Cd^2+^ content of WT and transgenic tobacco plants under cadmium stress. WT: wild-type; L1–L3: different transgenic lines.

Growth of both the transgenic and non-transgenic tobacco plants was similar before cadmium treatment. However, the growth of plants after cadmium stress could be seen to be obviously inhibited by cadmium, which greatly reduced the plant height, fresh weight, and relative chlorophyll content of the tobacco plants (Figure 3A–C,G). When compared with the wild-type seedlings, seedlings overexpressing *PsnMLP328* had a higher plant height, fresh weight, and relative chlorophyll content after cadmium treatment, and the percentage reductions in plant height and relative chlorophyll content were all significantly lower than those for the wild-type (Figure 3 and Figure 4). This indicates that overexpression of *PsnMLP328* can effectively alleviate the growth inhibition of tobacco by Cd^2+^ stress.

We subsequently determined the SOD activity, MDA content, and proline content of different tobacco lines before and after stress. The results showed that cadmium stress did not cause a significant change in the SOD activity in tobacco. However, the SOD activity of the transgenic lines was significantly higher than that of the wild-type lines after stress (Figure 3D). The trend for the MDA content was different to that for SOD activity, in that the MDA content of transgenic lines was significantly lower than that of the wild-type lines before stress, and cadmium stress caused a decrease in the MDA content of all lines, but there was no significant difference among the lines (Figure 3F). Similarly, Cd^2+^ stress caused a decrease in proline content in all the tobacco lines, with significant differences in proline content changes before and after stress in the wild-type lines but not in the transgenic lines, and all the transgenic lines had a higher proline content than the wild-type (Figure 3E).

To further investigate the molecular mechanism by which *PsnMLP328* regulates the tolerance of plants to cadmium stress, we measured the Cd^2+^ concentration in transgenic as well as non-transgenic tobacco plants after stress. However, the total amount of Cd^2+^ accumulated in the body of transgenic plants was more than that of non-transgenic plants since they had a larger biomass after Cd^2+^ stress (Figure 4). The results showed that the Cd^2+^ concentration in the transgenic tobacco plants was significantly lower than that in the wild-type.

From these results, it can be seen that overexpression of *PsnMLP328* can enhance the resistance of tobacco to both salt stress and cadmium stress, but the enhancement of tolerance in the transgenic plants was more obvious for cadmium stress compared with that of salt stress. Therefore, we focused on cadmium stress to further investigate the molecular mechanism by which *PsnMLP328* enhances tolerance to cadmium stress in plants.

### 2.4. Expression Analysis of Transgenic Plants and the Prediction of Genes Regulated by PsnMLP328

**Figure 5:**
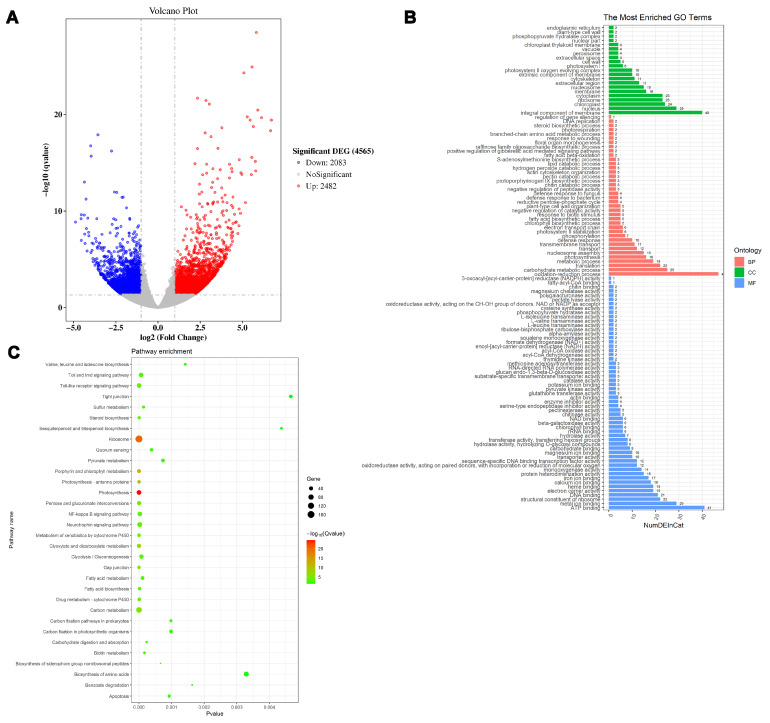
RNA-Seq analysis of transgenic tobacco plants. (**A**) Volcano plot of DE (differentially expressed) genes. (**B**) GO analysis of the DE genes. (**C**) KEGG analysis of the DE genes.

The analysis of differential expression in transgenic and non-transgenic tobacco plants using RNA-Seq showed that a total of 4565 genes were differentially expressed after overexpression of *PsnMLP328* in tobacco, of which 2482 genes were up-regulated and 2083 genes were down-regulated (Figure 5A). Subsequently, GO classification revealed that a total of 332 genes were annotated to the three major categories of biological processes, cellular components, and molecular functions. Among the molecular functions, there were most of the genes were involved in ion binding, ribosome structure composition, calcium-ion binding, and protein heterodimer activity. In the cellular component classification, most of the genes comprised membrane components, chloroplast components, ribosome components, and nucleosome components. In biological processes, the genes were mainly related to redox processes, carbohydrate metabolism, translation, photosynthesis, nucleosome assembly, transporter, stress responses, and other processes. KEGG enrichment analysis revealed that a total of 797 genes were involved in 21 KEGG pathways. Among them, the pathways in which the most differentially expressed genes were enriched were metabolic pathways, secondary metabolite synthesis pathways, and the ribosome pathway (Figure 5B,C).

Following further screening of the RNA-Seq results, it was noted that there were remarkable changes in the expression of some metal-ion transporters in the transgenic plants overexpressing *PsnMLP328*. The log2-fold change in the potassium ion channels *AKT1* and *AKT2/3* were 2.4 and 1.7 in transgenic plants, respectively. The log2-fold change of the heavy metal–nicotinamide transporter *YSL2*, which mediates Cd^2+^ transport from root to shoot, was 3.5, whereas the expression of *Nramp2-like* was -2.1 (Table 2).

**Figure 6:**
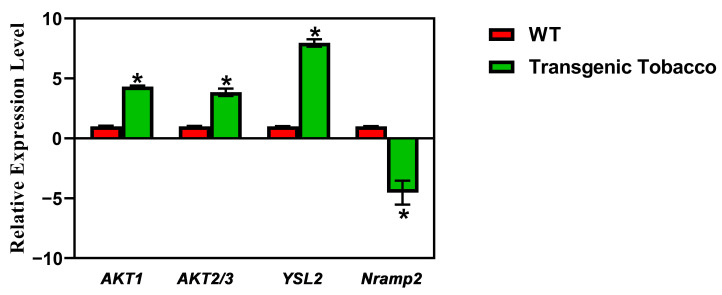
Real-time PCR of the candidate genes.* indicates a *p*-value < 0.05.

The results of real-time PCR also confirmed the accuracy of the RNA-Seq results (Figure 6). Therefore, we hypothesized that *PsnMLP328* might regulate the cadmium ion concentration and resistance to cadmium stress in plants by regulating the activities of various metal-ion transporters.

## 3. Discussion

Throughout their development, plants may encounter a range of external abiotic challenges, including heavy metal stress, cold stress, and salt stress [8]. Furthermore, plants, particularly tree species, have a greater potential for phytoremediation than herbaceous plants because of their deeper root systems and higher biomass, as well as the potential to enhance adaptations to allow them to thrive in stressful conditions [18,25,37]. *E. cammaldulesis* and *T. aphylla*, for example, may sustain a high K^+^/Na^+^ ratio under high levels of salt stress (30 dS^−1^) by lowering the K^+^ efflux and Na^+^ influx, making them more salt resistant and suitable for soil remediation [12]. Salt-tolerant hybrid poplar can be utilized to remediate soil and groundwater [38]; for example, *Acacia nilotica* L. has the ability to remediate Cd^2+^ metals due to its high stem biomass, stem uptake of Cd^2+^, and Cd^2+^ tolerance [39]. It is obvious that investigating the mechanisms by which trees cope with abiotic stress and improving their capacity to withstand them are crucial for the rehabilitation of contaminated soil.

Plant major latex protein is a member of the Bet_v_1 superfamily, which is a disease-resistance and growth- and development-related protein that plays a significant role in plant responses to pathogenic stressors as well as in growth and development [27]. Furthermore, in recent years, it has been suggested that members of the *MLP* family perform a role in the ways that plants react to abiotic stress, including to cold, salt, drought, and so on. The function of *MLP* genes in Cd^2+^ stress is as-yet unknown, which merits more investigation. Research on *MLP* family members in poplar and the potential mechanisms of *MLP* genes in response to abiotic stress have, however, hardly been reported. In this study, a gene encoding plant major latex protein, *PsnMLP328*, was cloned from *Populus simonii × P. nigra* and was studied by bioinformatics analysis, expression analysis under abiotic stress, and an analysis of downstream genes that it might regulate.

The results demonstrated that both salt stress and cadmium stress induced *PsnMLP328* expression. However, its expression level followed a pattern of an initial decrease, followed by an increase and then a subsequent decline over a prolonged stress duration. This phenomenon may be explained as follows: During the initial phase of salt stress, plants likely prioritize energy and resources for essential survival processes, such as cellular homeostasis and osmotic regulation, over gene transcription and translation. This leads to a temporary reduction in the expression of growth-related genes. As the stress persists, plants perceive stress signals and activate protective mechanisms, which require increased expression of stress-responsive genes, including *PsnMLP328.* Consequently, *PsnMLP328* expression rises and eventually stabilizes in the later stages. Tobacco lines overexpressing *PsnMLP328* exhibited enhanced resistance to both types of stress compared with the wild type. However, *PsnMLP328* appears to respond differently to the two types of stress, with a more pronounced ability to mitigate Cd^2+^-induced damage. The transgenic tobacco lines exhibited increased Cd^2+^ accumulation while maintaining higher biomass, elevated relative chlorophyll content, and reduced Cd^2+^ concentrations. RNA-Seq analysis revealed that *PsnMLP328* enhances tobacco resistance by boosting antioxidative enzyme activity and the proline content, reducing oxidative damage, and by regulating the expression of K^+^ and Cd^2^^+^ channels.

### 3.1. Possible Mechanisms by Which PsnMLP328 Regulates Salt-Stress Resistance in Plants

Malondialdehyde (MDA) is one of the end products of lipid peroxidation in organisms, which occurs when free radicals react with lipids and peroxide them [40]. Its levels might indicate the degree of lipid peroxidation damage in the organism or tissue, and it is a good indicator of oxidative stress [41]. Elevated MDA levels are frequently linked to oxidative stress and cellular damage. It has been demonstrated that when exposed to salt stress, plants that are more tolerant to the condition will have a lower MDA content than salt-sensitive types [42]. The current study’s findings indicate that tobacco plants overexpressing *PsnMLP328* have reduced MDA content when treated with salt, indicating that the transgenic tobacco plant has a higher tolerance to salt. Furthermore, when plants are exposed to environmental stress, particularly saline and alkaline stress, a large number of reactive oxygen species (ROS) accumulate, including superoxide anion radicals (O^2−^), hydrogen peroxide (H_2_O_2_), and singlet oxygen, among others, destroying membrane structures and interfering with normal plant metabolism via the peroxidation of nucleic acids, proteins, and lipids [43]. Additionally, ROS degrade biological membranes’ selectivity, increasing their permeability and causing membrane lipid peroxidation, all of which ultimately result in varying degrees of plant damage [44]. POD has multiple functions in plants. It is one of the key enzymes in the enzymatic defense system of plants in adverse conditions, and it works synergistically with superoxide dismutase (SOD) and catalase (CAT) to scavenge excess free radicals in the body, thereby improving plant resistance [27]. The superoxide content in plants increases after being exposed to external abiotic stress, while plants with stronger resistance have a lower accumulated superoxide content and a higher POD content than other plants, which enhances plant resistance under abiotic stress by alleviating the content of ROS in the body [45].

The results of this study show that tobacco plants overexpressing *PsnMLP328* have a lower reactive oxygen species content and a higher POD content, suggesting that the enhancement of salt-stress tolerance in plants by *PsnMLP328* may be achieved by directly or indirectly activating antioxidative enzymes, reducing the level of ROS, scavenging free radicals in the body, and protecting the plant from damage caused by reactive oxygen species. This finding is in line with earlier research showing that overexpressing *NtMLP43* can improve drought tolerance in tobacco plants and lessen the membrane damage and ROS buildup in plants under drought stress [34]. Another study also indicated that overexpression of *NtMLP423* reduced membrane lipid degradation, improved antioxidative enzyme activity, and decreased ROS generation during low-temperature stress, enhancing cold-stress resistance in tobacco [46].

The effects of salt stress on plants can be split into two categories: ion toxicity and osmotic stress [2]. Under salt stress, potassium ions in plants decrease while sodium ions increase, resulting in a reduced K^+^/Na^+^ ratio, which can lead to ionic toxicity or even plant death. For plants to withstand salt stress, it is crucial to maintain a comparatively high K^+^/Na^+^ ratio [3,47]. Interestingly, RNA-Seq analysis revealed that the transgenic lines had up-regulated expression of the K^+^ channels *AKT1* and *AKT2/3*. The *AKT* family is the first voltage-dependent K^+^ channel discovered in Arabidopsis, and it belongs to the Shaker K^+^ channel family [48]. *AKT1* is a key transporter for K^+^ uptake from the soil in plants, whereas *AKT2/3* is an inner rectifier-type K^+^ channel [49,50,51,52]. K^+^ channels have been linked to the preservation of a high K^+^/Na^+^ ratio in plants during salt stress [53]. Thus, we might hypothesize that *PsnMLP328* enhances tobacco resistance to salt stress by inducing the expression of the K^+^ channels *AKT1* and *AKT2/3*, which facilitates potassium ion uptake and transport and maintains the plant’s K^+^/Na^+^ ratio at a high level.

### 3.2. Possible Mechanisms by Which PsnMLP328 Regulates Cadmium-Stress Resistance in Plants

Cadmium stress has an impact on the morphological, biochemical, and physiological processes that influence plant growth. Previous research has demonstrated that Cd^2+^ stress decreases the root-crown length of wheat, pea, and blue bean (*Suaeda glauca*), as well as reducing maize and wheat dry weight [54,55,56,57,58]. The results of this investigation also showed that Cd^2+^ stress drastically inhibited tobacco plant growth, with all plants having significantly lower dry weight and plant height. However, tobacco plants overexpressing *PsnMLP328* had a higher dry weight and plant height than the wild-type, demonstrating that overexpression of *PsnMLP328* could improve resistance to Cd^2+^ stress. Like salt stress, Cd^2+^ stress can cause excessive ROS that are harmful to plant cells. For example, Dong et al. discovered that high concentrations of Cd^2+^ caused the generation and accumulation of ROS in the peanut cytoplasm, disrupting the integrity of the plasma membrane and the selective transport system and resulting in intracellular metal transport, which was harmful to the plants [59]. The current findings revealed that overexpressed *PsnMLP328* tobacco plants had significantly higher SOD levels than the wild-type. This suggests that *PsnMLP328* may aid plants in surviving Cd^2+^ stress by enhancing antioxidative enzyme activity and lowering ROS accumulation. Furthermore, the transgenic plants had a higher proline content when exposed to Cd^2+^, which has been related to plant responses to abiotic stresses such as heavy metal stress. Plants with a high proline content demonstrated greater Cd^2+^-stress tolerance [60,61]. As a result, *PsnMLP328* may aid plant survival by increasing the proline accumulation in response to Cd^2+^ stress.

As can be seen from Figure 3A, plant leaves under Cd^2+^ stress turn yellow, a symptom of a considerable reduction in chlorophyll content. Chlorophyll is a crucial component of plant photosynthesis, and its absence will result in a reduction in the plant biomass. In the current study, we discovered that after being exposed to Cd^2+^ stress, plants overexpressing *PsnMLP328* exhibited a higher biomass, a lower percentage drop in the biomass and chlorophyll content, and a higher chlorophyll content than the wild-type. Consequently, we believe that *PsnMLP328* is responsible for improving plants’ resistance to Cd^2+^ stress by lowering chlorophyll loss, raising the amount of chlorophyll in the plant following stress, preserving photosynthesis, and aiding the plant in gaining more biomass. 

Cadmium transport in plants involves the Nramp, HMA, ZIP, ATP, YSL, and ABC (ATP-Binding Cassette) families [19,62,63]. It was first demonstrated that the Nramp family was mostly engaged in the uptake and transport of Fe^2+^, whilst the YSL family encoded transporters of Fe^2+^ and Zn^2+^ [63,64]. Recent research, however, has shown that both families’ members are equally capable of absorbing and transporting Cd^2+^. According to Shintaro Koike’s research, Nramp2 is located at the vesicular membrane and has been linked to the accumulation of Cd^2+^ in rice, whereas OsYSL2 is primarily distributed in the rice phloem and plays a role in the phloem transport of Fe^2+^ and Mn^2+^ from underground to the shoot [64]. This finding implies that *PsnMLP328* might influence the expression of Cd^2+^ transporters in regulating the plant’s uptake and distribution of Cd^2+^, thereby assisting plants in surviving Cd^2+^ stress. Additionally, we observed in this work that overexpression of *PsnMLP328* led to a decrease in Cd^2+^ content but an increase in the total Cd^2+^ absorbed in plants when compared with the wild-type.

This finding is consistent with the results of a previous study, which discovered that overexpression of *PyWRKY75* significantly increased Cd^2+^ uptake and accumulation in *Populus yunnanensis*, with the amount of Cd^2+^ accumulated in the body increasing by 51.32% compared with the wild-type but with the concentration of Cd^2+^ becoming lower [65]. Based on the experimental results and previous research, we proposed that tobacco plants transfected with *PsnMLP328* are more tolerant to cadmium stress while absorbing more Cd^2+^, which could be attributed to the following reasons: 

1. It has been demonstrated that the quantity of K^+^ and Cd^2+^ are strongly correlated, and that increasing the amount of K^+^ in a plant can improve its uptake of Cd^2+^ [66,67,68]. In the current work, plants overexpressing *MLP328* exhibited considerably higher expression of the potassium channels *AKT1*, *AKT2/3*. Consequently, *PsnMLP328* could increase K^+^ uptake in plants, which would, in turn, increase the amount of Cd^2+^ accumulated in plants, thereby assisting plants in absorbing Cd^2+^ from soil. 2. Transgenic plants showed a significant up-regulation of the expression of *YSL2*, a transporter that mediates the transport of Cd^2+^ from the root to the shoot, and a significant down-regulation of the expression of *Nramp2*, a transporter that mediates the efflux of Cd^2+^ from vesicles to the cytoplasm. In addition to regulating Cd^2+^ redistribution by lowering the Cd^2+^ vesicle-to-cytoplasmic efflux to maintain a lower Cd^2+^ concentration, *PsnMLP328* promotes Cd^2+^ absorption, which causes transgenic plants to have a higher total Cd^2+^ content than the wild-type. This can protect plants from Cd^2+^ toxicity. 3. Chlorophyll content is critical for plant photosynthesis and biomass, and *PsnMLP328* can help plants retain a higher chlorophyll content under Cd^2+^ stress to assure a relatively large biomass, lowering the concentration of Cd^2+^ in the plant and mitigating Cd^2+^ toxicity.

These results suggest that overexpression of *PsnMLP328* can improve plants’ ability to tolerate Cd^2+^stress, increasing their biomass while additionally enabling them to absorb more Cd^2+^ from the soil, thus cleaning up cadmium-contaminated soils. The findings of this study provide theoretical support for the use of woody plants in the repair of heavy metal-ion-contaminated soil.

## 4. Materials and Methods

### 4.1. Plant Materials and Growth Conditions

The plant materials (*Populus simonii × P. nigra*) were provided by Northeast Forestry University. The wild-type tobacco plant (*Nicotiana tabacum* L. cv. Petit Havana SR-1) was used for genetic transformation. The poplar and tobacco seedlings were grown in 1/2 MS medium at 22 °C under a 16 h/8 h (light/dark) photoperiod and 75% relative humidity.

### 4.2. Gene Cloning and Sequence Analysis of PsnMLP328

Total RNA was extracted from the leaves of poplar (*Populus simonii × P. nigra*) using the RNAprep Pure Plant Kit (Tiangen, Beijing, China). A total of 1 μg of RNA was used for reverse transcription using a Takara PrimeScript™ RT reagent Kit with gDNA Eraser (Takara, Beijing, China). The cDNA was used as a template for PCR amplification using the high-fidelity enzyme KOD-plus-neo (Toyobo, Osaka, Japan) and specific primers, and the PCR products was recovered with the Tiangel purification kit (Tiangen, Beijing, China). The CDS (with terminator) of *PsnMLP328* was linked to the pGEM^®^-T vector (Promega, Beijing, China) and sequenced.

The ProtParam (https://web.expasy.org/protparam/) (accessed on 2 May 2024) software was used to analyze the physical and chemical properties of PsnMLP328. Signal-peptide analysis was performed using SignalP 5.0 (https://services.healthtech.dtu.dk/service.php?SignalP-5.0) (accessed on 2 May 2024) and Cell-PLoc 2.0 (http://www.csbio.sjtu.edu.cn/bioinf/Cell-PLoc-2/) (accessed on 2 May 2024) was used for the subcellular localization prediction. The transmembrane regions of proteins were predicted by cTMHMM Service 2.0 (https://services.healthtech.dtu.dk/service.php?TMHMM-2.0) (accessed on 2 May 2024). After searching for similarity sequences with the Blast program, the amino acid sequences of the same protein from different plants with high similarity to it were selected, and the multiple-sequence comparison and evolutionary trees were drawn with ClustalX1.83 and MEGA 5.0.

### 4.3. Vector Construction and Transformation of Nicotiana tabacum L. cv. Petit Havana SR-1

The CDS of *PsnMLP328* was inserted into the pROKII vector for transformation. The tobacco transformation and cultivation was based on the published literature, with some modifications [69]. The construct was transformed into the *Agrobacterium tumefaciens* strain AH105 and then transformed into SR-1 using the leaf-disc method. The leaves of infected tobacco plants were incubated on a substrate in the dark for 2 days, after which the buds were transferred into rooting medium with NAA to generate seedlings.

### 4.4. Identification of Transgenic Plants

Total DNA extraction was performed using the plant genomic DNA kit (Tiangen, Beijing, China), and total RNA was extracted using the RNAprep Pure Plant Kit (Tiangen, Beijing, China). PCR was used to identify transgenic plants, and real-time PCR was used to detect the level of overexpression.

### 4.5. Plant Treatment and Expression Analysis

Treatment and expression analysis of transgenic tobacco seedlings: Two-week-old T_2_-generation tobacco plants with similar growth were selected and subjected to stress treatment. Under normal conditions, plants were grown on 1/2 MS medium. For the salt-stress treatment, plants were grown on 1/2 MS medium supplemented with 150 mM NaCl, and for the cadmium-stress treatment, plants were grown on 1/2 MS medium supplemented with 100 μM CdCl_2_. On the 30th day after treatment, the fourth leaves of different plants were collected for physiological analysis, RNA-Seq analysis, and phenotypic observation (photography). Relative chlorophyll content was detected by a chlorophyll analyzer (SPAD502-PLUS, Konica Minolta, Tokyo, Japan). The POD content, SOD activity, MDA content, and proline content were detected using kits from Solarbio (Solarbio, Shanghai, China).

Transcriptome analysis: Total RNA was extracted from tobacco leaves using the RNAprep Pure Plant Kit (Tiangen, Beijing, China). Library construction and sequencing were performed by Genewiz Co., Ltd. (Suzhou, China), following Illumina’s standard protocol [70]. Gene expression levels were quantified using the transcripts-per-million (TPM) method via RSEM. Differential expression analysis used the DESeq2 Bioconductor package, a model based on the negative binomial distribution. The estimates of dispersion and logarithmic fold changes incorporate data-driven prior distributions, and the Padj values of the genes were set at ≤0.05 to detect differentially expressed ones. GOSeq (v1.34.1) was used to identify Gene Ontology (GO) terms to annotate the list of enriched genes with a significant Padj of ≤0.05, while topGO was used to plot the DAG. KEGG (Kyoto Encyclopedia of Genes and Genomes) is a collection of databases dealing with genomes, biological pathways, diseases, drugs, and chemical substances (http://en.wikipedia.org/wiki/KEGG) (accessed on 15 October 2024). We used in-house scripts to enrich for significantly differentially expressed genes in KEGG pathways.

Treatment and expression analysis of poplar: hydroponic poplar branches with similar growth were selected for stress treatment. The salt-stress treatment was 150 mM NaCl, and the cadmium-stress treatment was 100 μM CdCl_2_. Leaves were taken at 0, 6, 12, 24, 48, and 72 h time points, respectively, and the cDNA was extracted and then utilized for expression detection by realtime-PCR.

## 5. Conclusions

In this study, *PsnMLP328*, a member of the plant major latex protein family, has been cloned and analyzed preliminarily with regard to its physicochemical properties and evolution. Both salt stress and cadmium stress could induce the expression of *PsnMLP328*, but it had different response modes to the two stresses. Overexpression of *PsnMLP328* could enhance the resistance of tobacco to both salt and cadmium stress, but *PsnMLP328* could better alleviate the toxicity of cadmium stress on plants than that of salt stress. Based on the subsequent physiological and transcriptome analyses, we hypothesize that the mechanisms by which *PsnMLP328* elevates tobacco resistance to salt stress and cadmium stress are as follows:

1. *PsnMLP328* decreases oxidative damage in stressed plants by improving antioxidative enzyme activity and the proline concentration, hence aiding the plant’s adaptation to stress. 2. *PsnMLP328* overexpression increases the amount of chlorophyll in stressed plants and promotes their growth to withstand environmental stress. 3. *PsnMLP328* increases potassium levels in the body by up-regulating K^+^ channels and maintaining a higher K^+^/Na^+^ ratio, allowing plants to tolerate salt. 4. *PsnMLP328* enhances the K^+^ content by up-regulating the expression of K^+^ channels, which promotes the absorption of Cd^2+^ by plants. Regulating the Cd^2+^ influx and regionalized transporters decreases the Cd^2+^ concentration in plants while raising the uptake of Cd^2+^ by plants, which helps to relieve the Cd^2+^ stress.

In summary, it was determined that *PsnMLP328* has a favorable regulating effect in *Populus simonii × P. nigra* in response to salt stress and cadmium stress, particularly cadmium stress. Further investigation into the precise molecular pathways involved is worthwhile, since it holds significant importance for the bioremediation of salty and heavy metal soils when employing woody plants. The findings provide a strong molecular basis for enhancing polluted soil phytoremediation. In the meanwhile, the Cd^2+^ -rich transgenic lines produced with this gene show promise for use in the phytoremediation of soils contaminated with Cd^2+^.

## Figures and Tables

**Table 1:** Functional site analysis of PsnMLP328.

	Casein kinase II phosphorylation site
35–38:	Phosphoserine
106–109:	Phosphoserine
	N-glycosylation site
33–36:	N-linked (GlcNAc…) asparagine
79–82:	N-linked (GlcNAc…) asparagine
	Protein kinase C phosphorylation site
54–56:	Phosphoserine
97–99:	Phosphoserine
101–103:	Phosphothreonine

**Table 2:** RNA-Seq results of candidate genes.

Gene Name	Log_2_-Fold Change	*p* Value	Padj	Description
*AKT1*	2.4053718	4.21 × 10^−5^	0.001272395	potassium channel AKT1-like%2C transcript variant X2
*AKT2/3*	1.754015611	2.90 × 10^−6^	0.000163073	potassium channel AKT2/3-like
*YSL2*	3.514848502	1.58 × 10^−12^	7.44 × 10^−10^	metal–nicotianamine transporter YSL2-like%2C transcript variant X1
*Nramp2*	−2.125372581	0.000164615	0.003521159	metal transporter Nramp2-like

## Data Availability

The original contributions presented in this study are included in the article/Supplementary Materials. Further inquiries can be directed to the corresponding author.

## Supplementary Materials

The following supporting information can be downloaded at: https://www.mdpi.com/article/10.3390/ijms26073350/s1.

## References

[1] Khan S., Naushad M., Lima E.C., Zhang S., Shaheen S.M., Rinklebe J. (2021). Global soil pollution by toxic elements: Current status and future perspectives on the risk assessment and remediation strategies—A review. J. Hazard. Mater..

[2] Litalien A., Zeeb B. (2020). Curing the earth: A review of anthropogenic soil salinization and plant-based strategies for sustainable mitigation. Sci. Total Environ..

[3] van Zelm E., Zhang Y., Testerink C. (2020). Salt Tolerance Mechanisms of Plants. Annu. Rev. Plant Biol..

[4] Nakashima K., Ito Y., Yamaguchi-Shinozaki K. (2009). Transcriptional Regulatory Networks in Response to Abiotic Stresses in Arabidopsis and Grasses. Plant Physiol..

[5] Hasegawa P.M., Bressan R.A., Zhu J.K., Bohnert H.J. (2000). Plant cellular and molecular responses to high salinity. Annu. Rev. Plant Biol..

[6] Ma L., Liu X., Lv W., Yang Y. (2022). Molecular mechanisms of plant responses to salt stress. Front. Plant Sci..

[7] Yu Z., Duan X., Luo L., Dai S., Ding Z., Xia G. (2020). How plant hormones mediate salt stress responses. Trends Plant Sci..

[8] Qin G., Niu Z., Yu J., Li Z., Ma J., Xiang P. (2021). Soil heavy metal pollution and food safety in China: Effects, sources and removing technology. Chemosphere.

[9] Asare M.O., Száková J., Tlustoš P. (2023). Mechanisms of As, Cd, Pb, and Zn hyperaccumulation by plants and their effects on soil microbiome in the rhizosphere. Front. Environ. Sci..

[10] Lai C.W., Lou F., Liu J.L., Liu L., Zhou Y.Y., Shi S.Y. (2023). Progress and Prospects of Research on Cadmium Tolerance Genes in Plants. J. Org. Chem. Res..

[11] Li Y., Ding L., Zhou M., Chen Z., Ding Y., Zhu C. (2023). Transcriptional Regulatory Network of Plant Cadmium Stress Response. Int. J. Mol. Sci..

[12] He J., Ma C., Ma Y., Li H., Kang J., Liu T., Polle A., Peng C., Luo Z.B. (2013). Cadmium tolerance in six poplar species. Environ. Sci. Pollut. Res. Int..

[13] Clemens S., Aarts M.G.M., Thomine S., Verbruggen N. (2013). Plant science: The key to preventing slow cadmium poisoning. Trends Plant Sci..

[14] Singh P., Mitra P., Goyal T., Sharma S., Sharma P. (2021). Blood lead and cadmium levels in occupationally exposed workers and their effect on markers of DNA damage and repair. Environ. Geochem. Health.

[15] Kumar S., Sharma A. (2019). Cadmium toxicity: Effects on human reproduction and fertility. Rev. Environ. Health.

[16] Noor I., Sohail H., Akhtar M.T., Cui J., Lu Z., Mostafa S., Hasanuzzaman M., Hussain S., Guo N., Jin B. (2024). From stress to resilience: Unraveling the molecular mechanisms of cadmium toxicity, detoxification and tolerance in plants. Sci. Total Environ..

[17] Niu L., Li C., Wang W., Zhang J., Scali M., Li W., Liu H., Tai F., Hu X., Wu X. (2023). Cadmium tolerance and hyperaccumulation in plants—A proteomic perspective of phytoremediation. Ecotoxicol. Environ. Saf..

[18] Yue C., Huang S.Y., Tu C.B., Wu C.F., He Y., Wang Z. (2024). Research Progress on Phytoremediation of Soil Contaminated by Heavy Metal Cadmium. Mod. Agric. Sci. Technol..

[19] Raza A., Habib M., Kakavand S.N., Zahid Z., Zahra N., Sharif R., Hasanuzzaman M. (2020). Phytoremediation of Cadmium: Physiological, Biochemical, and Molecular Mechanisms. Biology.

[20] Cao Y., Tan Q., Zhang F., Ma C., Xiao J., Chen G. (2022). Phytoremediation potential evaluation of multiple Salix clones for heavy metals (Cd, Zn and Pb) in flooded soils. Sci. Total Environ..

[21] Qiao K., Shan Q., Zhang H., Lv F., Zhou A. (2023). *Populus euphratica* plant cadmium tolerance PePCR3 improves cadmium tolerance. Tree Physiol..

[22] Shi R., Liang L., Liu W., Zeb A. (2022). *Kochia scoparia* L., a newfound candidate halophyte, for phytoremediation of cadmium-contaminated saline soils. Environ. Sci. Pollut. Res. Int..

[23] Hussain M.S., Naeem M.S., Tanvir M.A., Nawaz M.F., Abd-Elrahman A. (2021). Eco-physiological evaluation of multipurpose tree species to ameliorate saline soils. Int. J. Phytoremediat..

[24] Zhang Y., Sa G., Zhang Y., Hou S., Wu X., Zhao N., Zhang Y., Deng S., Deng C., Deng J. (2021). *Populus euphratica* annexin1 facilitates cadmium enrichment in transgenic Arabidopsis. J. Hazard. Mater..

[25] Bedair H., Ghosh S., Abdelsalam I.M., Keerio A.A., AlKafaas S.S. (2022). Potential implementation of trees to remediate contaminated soil in Egypt. Environ. Sci. Pollut. Res. Int..

[26] He J., Li H., Luo J., Ma C., Li S., Qu L., Gai Y., Jiang X., Janz D., Polle A. (2013). A Transcriptomic Network Underlies Microstructural and Physiological Responses to Cadmium in *Populus × canescens*. Plant Physiol..

[27] Fujita K., Inui H. (2021). Review: Biological functions of major latex-like proteins in plants. Plant Sci. Int. J. Exp. Plant Biol..

[28] Nessler C.L., Kurz W.G.W., Pelcher L.E. (1990). Isolation and analysis of the major latex protein genes of opium poppy. Plant Mol. Biol..

[29] Ipsen H., Løwenstein H. (1983). Isolation and immunochemical characterization of the major allergen of birch pollen (*Betula verrucosa*). J. Allergy Clin. Immunol..

[30] Inui H., Sawada M., Goto J., Yamazaki K., Kodama N., Tsuruta H., Eun H. (2013). A Major Latex-Like Protein Is a Key Factor in Crop Contamination by Persistent Organic Pollutants. Plant Physiol..

[31] Nawrot R., Lippmann R., Matros A., Musidlak O., Nowicki G., Mock H.P. (2017). Proteomic comparison of *Chelidonium majus* L. latex in different phases of plant development. Plant Physiol. Biochem..

[32] Holmquist L., Dölfors F., Fogelqvist J., Cohn J., Kraft T., Dixelius C. (2020). Major latex protein-like encoding genes contribute to *Rhizoctonia solani* defense responses in sugar beet. Mol. Genet. Genom..

[33] Song L., Wang J., Jia H., Kamran A., Qin Y., Liu Y., Hao K., Han F., Zhang C., Li B. (2020). Identification and functional characterization of *NbMLP28*, a novel MLP-like protein 28 enhancing Potato virus Y resistance in *Nicotiana benthamiana*. BMC Microbiol..

[34] Wang Y., Yang L., Chen X., Ye T., Zhong B., Liu R., Wu Y., Chan Z. (2016). Major latex protein-like protein 43(MLP43) functions as a positive regulator during abscisic acid responses and confers drought tolerance in *Arabidopsis thaliana*. J. Exp. Bot..

[35] Liu H., Ma X., Liu S., Du B., Cheng N., Wang Y., Zhang Y. (2020). The *Nicotiana tabacum* L. major latex protein-like protein 423 (NtMLP423) positively regulates drought tolerance by ABA-dependent pathway. BMC Plant Biol..

[36] Yao W.J., Wang L., Zhou B.R., Wang S.J., Li R.H., Jiang T.B. (2016). Over-expression of poplar transcription factor *ERF76* gene confers salt tolerance in transgenic tobacco. J. Plant Physiol..

[37] Manikandan M., Kannan V., Mahalingam K., Vimala A., Chun S. (2016). Phytoremediation potential of chromium-containing tannery effluent-contaminated soil by native Indian timber-yielding tree species. Prep. Biochem. Biotechnol..

[38] Landmeyer J.E., Rock S., Freeman J.L., Nagle G., Samolis M., Levine H., Cook A.M., O’Neill H. (2020). Phytoremediation of slightly brackish, polycyclic aromatic hydrocarbon-contaminated groundwater from 250 ft below land surface: A pilot-scale study using salt-tolerant, endophyte-enhanced hybrid poplar trees at a Superfund site in the Central Valley of California, April–November 2019. Remediat. J..

[39] Shabir R., Abbas G., Saqib M., Shahid M., Shah G.M., Akram M., Niazi N.K., Naeem M.A., Hussain M., Ashraf F. (2018). Cadmium tolerance and phytoremediation potential of acacia (*Acacia nilotica* L.) under salinity stress. Int. J. Phytoremediat..

[40] Ebone L.A., Caverzan A., Chavarria G. (2019). Physiologic alterations in orthodox seeds due to deterioration processes. Plant Physiol. Biochem..

[41] Han D., Han J., Xu T., Li T., Yao C., Wang Y., Luo D., Yang G. (2021). Isolation and preliminary functional characterization of *MxWRKY64*, a new WRKY transcription factor gene from *Malus xiaojinensis* Cheng et Jiang. In Vitr. Cell. Dev. Biol. Plant.

[42] Wei Z., Ye J., Zhou Z., Chen G., Meng F., Liu Y. (2021). Isolation and characterization of PoWRKY, an abiotic stress-related WRKY transcription factor from *Polygonatum odoratum*. Physiol. Mol. Biol. Plants.

[43] Wu B., Qi F., Liang Y. (2023). Fuels for ROS signaling in plant immunity. Trends Plant Sci..

[44] Mittler R., Zandalinas S.I., Fichman Y., Van Breusegem F. (2022). Reactive oxygen species signalling in plant stress responses. Nat. Rev. Mol. Cell Biol..

[45] Czégény G., Rácz A. (2023). Phenolic peroxidases: Dull generalists or purposeful specialists in stress responses. J. Plant Physiol..

[46] Liu H., Du B., Ma X., Wang Y., Cheng N., Zhang Y. (2023). Overexpression of major latex protein 423 (*NtMLP423*) enhances the chilling stress tolerance in *Nicotiana tabacum*. Plant Sci..

[47] Shabala S., Cuin T.A. (2008). Potassium transport and plant salt tolerance. Physiol. Plant..

[48] Lebaudy A., Véry A.A., Sentenac H. (2007). K+ channel activity in plants: Genes, regulations and functions. FEBS Lett..

[49] Hirsch R.E., Lewis B.D., Spalding E.P., Sussman M.R. (1998). A role for the AKT1 potassium channel in plant nutrition. Science.

[50] Lagarde D., Basset M., Lepetit M., Conejero G., Gaymard F., Astruc S., Grignon C. (1996). Tissue-specific expression of Arabidopsis *AKT1* gene is consistent with a role in K+ nutrition. Plant J. Cell Mol. Biol..

[51] Sandmann M., Skłodowski K., Gajdanowicz P., Michard E., Rocha M., Gomez-Porras J.L., González W., Corrêa L.G.G., Ramírez-Aguilar S.J., Cuin T.A. (2011). The K+ battery-regulating Arabidopsis K+ channel AKT2 is under the control of multiple post-translational steps. Plant Signal. Behav..

[52] Cuin T., Dreyer I., Michard E. (2018). The Role of Potassium Channels in *Arabidopsis thaliana* Long Distance Electrical Signalling: AKT2 Modulates Tissue Excitability While GORK Shapes Action Potentials. Int. J. Mol. Sci..

[53] Tian Q., Shen L., Luan J., Zhou Z., Guo D., Shen Y., Jing W., Zhang B., Zhang Q., Zhang W. (2021). Rice shaker potassium channel OsAKT2 positively regulates salt tolerance and grain yield by mediating K+ redistribution. Plant Cell Environ..

[54] Zhang X., Li M., Yang H., Li X., Cui Z. (2018). Physiological responses of *Suaeda glauca* and *Arabidopsis thaliana* in phytoremediation of heavy metals. J. Environ. Manag..

[55] Li L.Z., Tu C., Peijnenburg W.J.G.M., Luo Y.M. (2017). Characteristics of cadmium uptake and membrane transport in roots of intact wheat (*Triticum aestivum* L.) seedlings. Environ. Pollut..

[56] Rahman M.F., Ghosal A., Alam M.F., Kabir A.H. (2017). Remediation of cadmium toxicity in field peas (*Pisum sativum* L.) through exogenous silicon. Ecotoxicol. Environ. Saf..

[57] Kaya C., Okant M., Ugurlar F., Alyemeni M.N., Ashraf M., Ahmad P. (2019). Melatonin-mediated nitric oxide improves tolerance to cadmium toxicity by reducing oxidative stress in wheat plants. Chemosphere.

[58] Lian J., Zhao L., Wu J., Xiong H., Bao Y., Zeb A., Tang J., Liu W. (2020). Foliar spray of TiO2 nanoparticles prevails over root application in reducing Cd accumulation and mitigating Cd-induced phytotoxicity in maize (*Zea mays* L.). Chemosphere.

[59] Dong Y.J., Chen W.F., Liu F.Z., Wan Y.S. (2019). Physiological responses of peanut seedlings to exposure to low or high cadmium concentration and the alleviating effect of exogenous nitric oxide to high cadmium concentration stress. Plant Biosyst. Int. J. Deal. All Asp. Plant Biol..

[60] Siripornadulsil S., Traina S., Verma D.P.S., Sayre R.T. (2002). Molecular Mechanisms of Proline-Mediated Tolerance to Toxic Heavy Metals in Transgenic Microalgae. Plant Cell.

[61] Sun R.L., Zhou Q.X., Sun F.H., Jin C.X. (2007). Antioxidative defense and proline/phytochelatin accumulation in a newly discovered Cd-hyperaccumulator, *Solanum nigrum* L.. Environ. Exp. Bot..

[62] Tan L., Zhu Y., Fan T., Peng C., Wang J., Sun L., Chen C. (2019). OsZIP7 functions in xylem loading in roots and inter-vascular transfer in nodes to deliver Zn/Cd to grain in rice. Biochem. Biophys. Res. Commun..

[63] Zhao J., Yang W., Zhang S., Yang T., Liu Q., Dong J., Fu H., Mao X., Liu B. (2018). Genome-wide association study and candidate gene analysis of rice cadmium accumulation in grain in a diverse rice collection. Rice.

[64] Koike S., Inoue H., Mizuno D., Takahashi M., Nakanishi H., Mori S., Nishizawa N.K. (2004). OsYSL2 is a rice metal-nicotianamine transporter that is regulated by iron and expressed in the phloem. Plant J..

[65] Wu X., Chen Q., Chen L., Tian F., Chen X., Han C., Mi J., Lin X., Wan X., Jiang B. (2022). A WRKY transcription factor, PyWRKY75, enhanced cadmium accumulation and tolerance in poplar. Ecotoxicol. Environ. Saf..

[66] He Y., Liu M., Wang R., Salam M., Yang Y., Zhang Z., He Q., Hu X., Li H. (2021). Potassium regulates cadmium toxicity in *Microcystis aeruginosa*. J. Hazard. Mater..

[67] Liu P., Jin Z., Dai C., Guo L., Cui X., Yang Y. (2021). Potassium enhances cadmium resistance ability of *Panax notoginseng* by brassinolide signaling pathway-regulated cell wall pectin metabolism. Ecotoxicol. Environ. Saf..

[68] Huang B., Liao Q., Fu H., Ye Z., Mao Y., Luo J., Wang Y., Yuan H., Xin J. (2023). Effect of potassium intake on cadmium transporters and root cell wall biosynthesis in sweet potato. Ecotoxicol. Environ. Saf..

[69] Topping J.F. (1998). Tobacco transformation. Methods Mol. Biol..

[70] Kamitani M., Kashima M., Tezuka A., Nagano A.J. (2019). Lasy-Seq: A High-throughput library preparation method for RNA-Seq and its application in the analysis of plant responses to fluctuating temperatures. Sci. Rep..

