# Supplementary material for: A Major Latex Protein-Encoding Gene from Populus simonii × P. nigra (PsnMLP328) Contributes to Defense Responses to Salt and Cadmium Stress
Source: Int J Mol Sci. 2025 Apr 3;26(7):3350. doi: 10.3390/ijms26073350 (PMC11989736; doi:10.3390/ijms26073350)
Supplement: Supplementary file 1 [file ijms-26-03350-s001.zip › ijms-3487567-supplementary.pdf]

Table S1. Physical and chemical properties of *PsnMLP328*.

Molecular Weight(K Da)	Theoretical isoelectric point	Instability Index	Negatively Charged Residues(n)	Positively Charged Residues(n)	Subcellular Localization Prediction
16.77	5.09	21.32	25	17	cytoplasm

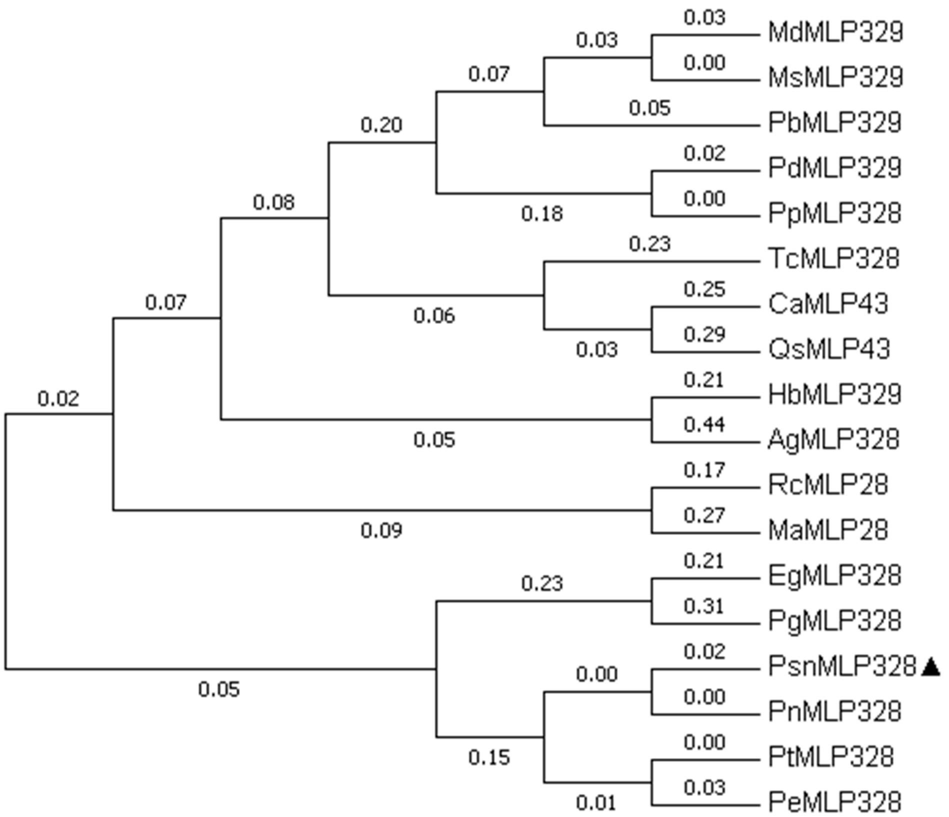

Figure S1 Phylogenetic analysis of PsnMLP328

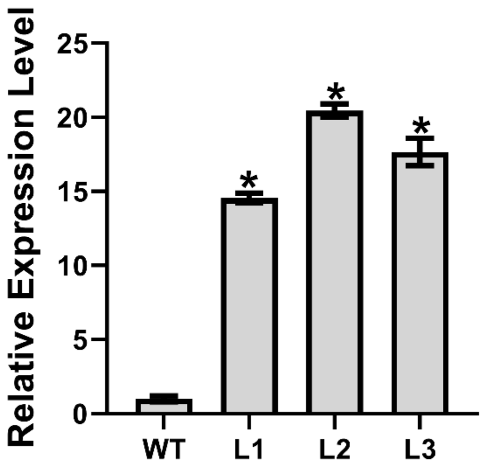

Figure S2 qRT-PCR result of transgenic tobaccos

WT: wild type, L1-L3: Tobacco lines that overexpress *PsnMLP328*,* indicates a p-value < 0.05
